# Neuronal involvement in muscular atrophy

**DOI:** 10.3389/fncel.2014.00405

**Published:** 2014-12-10

**Authors:** Bruno A. Cisterna, Christopher Cardozo, Juan C. Sáez

**Affiliations:** ^1^Departamento de Fisiología, Pontificia Universidad Católica de ChileSantiago, Chile; ^2^Center of Excellence for the Medical Consequences of Spinal Cord Injury, James J. Peters Veterans Affairs Medical CenterBronx, NY, USA; ^3^Departments of Medicine and Rehabilitation Medicine, Icahn School of Medicine at Mount SinaiNew York, NY, USA; ^4^Instituto Milenio, Centro Interdisciplinario de Neurociencias de Valparaíso, Universidad de ValparaísoValparaíso, Chile

**Keywords:** acetylcholine, hemichannels, connexins, trophic factors, electrical activity

## Abstract

The innervation of skeletal myofibers exerts a crucial influence on the maintenance of muscle tone and normal operation. Consequently, denervated myofibers manifest atrophy, which is preceded by an increase in sarcolemma permeability. Recently, *de novo* expression of hemichannels (HCs) formed by connexins (Cxs) and other none selective channels, including P2X_7_ receptors (P2X_7_Rs), and transient receptor potential, sub-family V, member 2 (TRPV2) channels was demonstrated in denervated fast skeletal muscles. The denervation-induced atrophy was drastically reduced in denervated muscles deficient in Cxs 43 and 45. Nonetheless, the transduction mechanism by which the nerve represses the expression of the above mentioned non-selective channels remains unknown. The paracrine action of extracellular signaling molecules including ATP, neurotrophic factors (i.e., brain-derived neurotrophic factor (BDNF)), agrin/LDL receptor-related protein 4 (Lrp4)/muscle-specific receptor kinase (MuSK) and acetylcholine (Ach) are among the possible signals for repression for connexin expression. This review discusses the possible role of relevant factors in maintaining the normal functioning of fast skeletal muscles and suppression of connexin hemichannel expression.

## Introduction

The control of skeletal muscle function by the nervous system has been of interest to researchers for more than 100 years in studies examining diverse aspects from the effects of mechanical loading to functions of specific molecular signals (Baldwin et al., 2013). The nervous system exerts control over skeletal muscles by two mechanisms: (1) neuromotor control, by which muscle contraction is initiated by nerve impulses generated in the brain cortex or the brainstem, depolarization of the sarcolemma and electromechanical coupling; and (2) neurotrophic control, which is independent of the electrical activity of motoneurons, and depends on the release of soluble factors from the nerve terminals of motor neurons at the neuromuscular junction (NMJ).

The importance of neural influences on skeletal muscle is evident from the rapid and severe muscular atrophy that occurs whenever there is loss of neural continuity (e.g., due to CNS injury, or the transection or compression of a nerve) (Tomanek and Lund, 1973; Zeman et al., 2009); the ensuing atrophy is considerably more rapid than that from other etiologies such as immobilization (Tomanek and Lund, 1974), cachexia (Dell, 2002; Tisdale, 2002), malnutrition (Morley, 2012), severe burns (Wu et al., 2010), aging (Demontis et al., 2013), dystrophies (Rahimov and Kunkel, 2013), and myasthenia gravis (Keesey, 2004; Ishii et al., 2005). Muscle atrophy results in great extent from accelerated turnover of proteins by the ubiquitin-proteasome pathway, often coupled with diminished rates of protein synthesis (Glass, 2003). Critical roles for signaling by myostatin, NF-kB, and FoxO1 and FoxO3A have been defined and have been reviewed in detail (Rüegg and Glass, 2011; Jackman et al., 2013).

Application of electrical stimulation to nerves to elicit muscle contractions can prevent or largely reverse muscle wasting due to paralysis indicating the critical role of muscle contraction in suppressing the signaling responsible for muscle atrophy (Dudley-Javoroski and Shields, 2008; Kim et al., 2008). There is also a vital role for presence of an intact lower motor neuron and NMJ, as demonstrated by findings of slowed muscle atrophy after spinal isolation (a variant of spinal cord injury, SCI) as compared to nerve transection (Hyatt et al., 2003). On the other hand, denervated muscle after temporary sensory nerve innervation, which provides support to the denervated muscle, improves functional recovery (Bain et al., 2001; Zhao et al., 2004).

One consequence of nerve transection is increased membrane permeability, reduced membrane potential, and increased membrane excitability. Most of these changes have recently been proposed to result from the *de novo* synthesis and insertion of connexin 39, 43 and 45 channels into the sarcolemma, which in turn have been found to mediate atrophy of fast skeletal muscle (Cea et al., 2013). This review compiles and discusses the information on the influence of the nervous system on skeletal muscles and their atrophy, and introduces the current state of knowledge regarding mechanisms by which the nervous system regulates skeletal muscle and its function.

## Muscular atrophy induced by denervation

When muscle is denervated due to injury of lower motor neurons there ensues a flaccid paralysis and rapid atrophy with reduction in muscle mass, strength and myofiber diameter; apoptosis of myofiber occurs (Siu and Alway, 2005) together with loss of muscle fibers (Tews, 2002). Most reports indicate that as early as 7 days post-denervation there is a significantly decreased diameter of myofibers in mice (Bruusgaard and Gundersen, 2008; Cea et al., 2013), rats (Pellegrino and Franzini, 1963) and guinea-pigs (Tomanek and Lund, 1973).

It is well documented that the axonal stump that remains after nerve injury undergoes a degenerative process known as Wallerian degeneration (Salzer and Bunge, 1980). However, the axonal stump maintains some physiological activity on skeletal muscles for up to 1 day. Pioneering investigations in denervation showed that there is a direct relationship between the length of the axonal stump and time course of failure of the stump to transmit impulses to the muscle (Eyzaguirre et al., 1952; Gutmann et al., 1955; Birks et al., 1960). The axonal stump was demonstrated to retain the ability to generate spontaneous miniature end-plate potentials (MEPPs) and end-plate potentials (EPPs) that evoke muscle contraction for 8–10 h. Failure of the stump to generate MEPPs is preceded by a gradual decrease in their frequency, while EPPs fail abruptly (Miledi and Slater, 1970). In addition, it was established that the ability of the stump to transmit impulses is prolonged by about 45 min for each additional centimeter in the axonal stump, suggesting that there is a direct relationship between the length of the axonal stump and the transmission of impulses to the muscle (Miledi and Slater, 1970). Similarly, the axonal stump length also influences the onset of muscular disorders such as fibrillation and hypersensitivity to acetylcholine (Ach; Luco and Eyzaguirre, 1955). This finding suggests that there is transport and release of factor(s) from the axonal stump, which ultimately are depleted as axonal reserves are consumed by the myofibers. This idea was strengthened with a clinical observation of weakness and muscle atrophy after accidental overdose of vincristine, which blocks axonal transport (Maeda et al., 1987). Together, these observations indicate need for renewal of the synaptic machinery of the nerve endings responsible for the MEPPs and EPPs.

The onset of fibrillation potentials in denervated muscles (Luco and Eyzaguirre, 1955; Hník and Skorpil, 1962), usually coincides with the reduction of resting membrane potential (Ware et al., 1954; Thesleff, 1963; Albuquerque and Thesleff, 1968), and was one of the first changes described; it has been suggested that fibrillation is the result of membrane depolarization (Ware et al., 1954; Li, 1960; Gage et al., 1989). However, there is no conclusive evidence about the origin of these alterations or their interrelationship.

The membrane depolarization after denervation is associated with several changes in ion current, permeability and concentrations. During the first post-denervation week, there is an increase in intracellular Na^+^ concentration, a decrease in intracellular K^+^ concentration, and an increase in total calcium content, as well as increased Na^+^ permeability (*P*_Na+_) and Na^+^ conductance, and decreased *P_K+_* (Purves and Sakmann, 1974b; Picken and Kirby, 1976; Smith and Thesleff, 1976; Kotsias and Venosa, 2001). This can be explained in part by the massive expression of ion channels such as cardiac-type voltage-gated Na^+^ channels (Sekiguchi et al., 2012), fetal acetyl choline receptor (Emmanouilidou et al., 2010) isoform and associated hypersensitivity to ACh (Rosenblueth and Luco, 1937), the tetrodotoxin (TTX) Na^+^-resistant channels (Harris and Thesleff, 1971), hemichannels (HCs) formed by connexins (Cxs 39, 43, and 45; Cx HCs), pannexin1 (Panx1) channels, purinergic ionotropic P2X_7_ receptors (P2X_7_Rs), transient receptor potential, sub-family V, member 2 (TRPV2), all of which are channels that are found at high levels in the sarcolemma within the first week after denervation (Cea et al., 2013). There is also increased expression of the cardiac Ca^2+^ permeable dihydropyridine receptor isoform, but this occurs at 25 days post-denervation (Péréon et al., 1997), and thus is not directly related to the onset of resting membrane potential decay. Recently, it was demonstrated that absence of two Cxs (43 and 45) significantly decreased the loss of myofiber size in muscles studied at day 7 post-denervation and prevented activation of the p65 subunit of NF-kB and up regulation of pro-inflammatory cytokines (TNF-α and IL-1β) (Cea et al., 2013). This finding raises the question of whether *de novo* expression of Cxs is an upstream response to many of the changes observed in myofibers after denervation. If so, their expression and activation might be somehow regulated by the innervation state or activity of the myofibers.

## Muscular atrophy induced by spinal cord injury

The disruption of continuity of the nervous system at the level of upper motor neurons may occur as a consequence of neurological conditions such as stroke, multiple sclerosis, or injury to the spinal cord (SCI) and acutely results in paralysis and atrophy of muscles; in SCI, affected muscles are those innervated by motor neurons arising from spinal cord segments below the level of the injury (Shields and Dudley-Javoroski, 2007; Qin et al., 2010). These neurological conditions result in diverse abnormalities, including spastic paralysis (Maynard et al., 1990; Sköld et al., 1999), weakness (Thomas et al., 1997), and extensor plantar responses. In SCI the lower motor neurons remain intact (Kaelan et al., 1988; Bjugn et al., 1997) but deterioration of motor neuron arborization and motor endplates occurs. Very heterogeneous NMJ subgroups (pre and post synaptic) are observed, with some present massive sprouting of nerve terminals, some loss of concentrated clusters of ACh receptors (AChRs) and others remaining intact (Burns et al., 2007); neuromuscular transmission is impaired (Ollivier-Lanvin et al., 2009). While denervation rapidly results in flaccid paralysis and late fibrillation, SCI initially presents as spinal shock and flaccid paralysis and is followed by the development over a period of several weeks or more of hyperreflexia and spasticity (Ditunno et al., 2004; Harris et al., 2006). Following SCI there ensues a brisk and extensive atrophy of skeletal muscle in mice, rats, and humans (Qin et al., 2010). In rodents, transection of the spinal cord, which results in complete loss of volitional activation of motor neurons arising below the anatomical level of the SCI, causes hindlimb muscle atrophy by as much as 40–60% (Ung et al., 2010; Wu et al., 2012). Similarly, muscle biopsy studies in humans suggest that following SCI, muscle fibers atrophy by 27 to 56% within the first 6–18 months after injury (Castro et al., 1999). These changes are associated with marked reductions in contractile force and fatigue resistance, loss of slow- and fast-twitch oxidative fibers, and diminished levels of enzymes for oxidative phosphorylation (Qin et al., 2010). It has been shown that in muscle from SCI rats studied at 56 days after the onset of paralysis, there are elevated sarcolemmal levels of Cxs 39, 43 and 45, and Panx 1 (Cea et al., 2013), which as noted above stimulates activation of the p65 subunit of NF-kB and drives muscle atrophy in muscle paralyzed by nerve transection. These findings suggest that elevation of membrane hemichannel expression may be involved in initiating muscle atrophy after SCI, and other neurological disorders that spare lower motor neurons such as stroke or multiple sclerosis.

## Electrical stimulation reverses muscle atrophy

Evidence supporting the view that the lack of neuromotor activity is responsible for the characteristics of muscle atrophy comes from the experiments with electrical stimulation (Hník et al., 1962; Salmons et al., 2005; Adami et al., 2007). After denervation, the early initiation functional electrical stimulation (FES) reverses the fibrillation potentials (Jones and Vrbová, 1970; Purves and Sakmann, 1974a), ACh hypersensitivity (Lomo and Rosenthal, 1972; Lomo and Westgaard, 1975), and TTX-resistant Na^+^ channel expression (Award et al., 1965). However, it does not prevent loss of membrane potential (Squecco et al., 2009). In SCI, loss of muscle mass is also reversed by FES (Scremin et al., 1999). When used for extended periods, FES greatly increases muscle volume, strength and endurance, and the expression of slow myosin heavy chain isoforms (Dudley-Javoroski and Shields, 2008; Qin et al., 2010). It was recently demonstrated that muscle responds rapidly to FES with the greatest number of gene expression changes occurring within the first 1–3 days after initiating electrical stimulation (Ma et al., 2007; Wu et al., 2013). Genes regulated by FES included those for nicotinic AChRs (Adams and Goldman, 1998; Wu et al., 2013), suggesting remodeling of NMJs, possibly to support more efficient neuromuscular transmission. A striking difference between the effects of FES on chronically paralyzed muscle of rats or humans, and physical activity in the absence of SCI is the delayed and impaired upregulation of genes, supporting oxidative phosphorylation in response to FES (Rochester et al., 1995; Wu et al., 2013). Whether this reflects an effect of a non-physiologic pattern of neural and neuromuscular activation, improperly organized signaling molecules at the NMJ, or impaired mechanisms downstream of AChR activation, such as absence of key transcription factors or slowly reversible epigenetic modifications, is unclear. Eventually, more normal gene expression responses are induced in humans by FES, suggesting that the deficit, regardless of its cause, is reversible. Intriguingly, even brief periods of FES are sufficient to prevent muscle atrophy after SCI (Baldi et al., 1998; Kim et al., 2008), suggesting that ACh actions on skeletal muscle persist long after the membrane depolarization and contraction have ceased. The nature of these persistent effects remains unclear.

## Connexins and skeletal muscle

Connexins are membrane proteins that form poorly selective channels in the cellular membrane that are also called HCs or connexons. Classically, an HC forms an axially aligned complex with another HC present in the membrane of an adjacent cell to form an intercellular pore (gap junction channel), which directly connects the cytoplasm of adjoining cells (Sáez et al., 2005). More recently, HCs have been found to connect the intra and extra-cellular compartments, allowing transfer of ions such as Na^+^ (Li et al., 2001), K^+^ (Wallraff et al., 2006), and Ca^2+^ (Li et al., 2001; Sánchez et al., 2009; Schalper et al., 2010; Fiori et al., 2012), entry of nutrients such as glucose (Retamal et al., 2007), release of metabolic products such as glutathione (Rana and Dringen, 2007), as well as of autocrine and paracrine signals such as ATP (Stout et al., 2002), NAD^+^ (Bruzzone et al., 2001), cADPR (Song et al., 2011), IP_3_ (Gossman and Zhao, 2008), glutamate (Ye et al., 2003), and prostaglandin E_2_ (Cherian et al., 2005).

Myoblasts express Cxs and form gap junctions that are essential for development during early and late stages of myogenesis (Constantin and Cronier, 2000). These gap junctions are likely to coordinate gene expression and metabolic responses among differentiating myoblasts (Kalderon et al., 1977; Dennis et al., 1981; Constantin and Cronier, 2000; Araya et al., 2003, 2004; von Maltzahn et al., 2004; Belluardo et al., 2005). In the terminal stage of myogenesis there is down regulation of Cx expression (Armstrong et al., 1983; Proulx et al., 1997; Constantin and Cronier, 2000), and the progressive decline of electrical coupling between myofibers (Dennis et al., 1981; Ling et al., 1992). Connexins are absent in normal skeletal muscle fibers but they have been detected in myofibers of adult muscles undergoing regeneration after injury (Araya et al., 2004; von Maltzahn et al., 2004; Belluardo et al., 2005) and in the sarcolemma of muscle fibers at 7 days post-denervation or 56 days after SCI (Cea et al., 2013). Studies of denervation atrophy in mice deficient for skeletal muscle Cx43 and Cx45 have also demonstrated important roles for these HCs in the signaling through which atrophy occurs. As noted above, the double knockout reduced denervation atrophy by ~70% for fast muscles at 7 days associated with complete inhibition of the activation of the p65 subunit of NF-kB, which as mentioned above, has been shown to be a critical regulator of denervation atrophy. To summarize, Cxs are expressed during myogenesis, when muscle cells are not innervated, disappear within few days after birth, when muscle cells are innervated, rapidly emerge after denervation or paralysis due to upper motor neuron injury, and mediate key signals responsible for denervation atrophy.

These findings strongly suggest that innervation and/or neuromuscular activity suppress Cx expression in the sarcolemma of adult. The mechanism(s) that controls the expression of Cxs in skeletal muscle is, however, unknown, and the only existing evidence relates to the process of myogenesis and points to microRNAs (miRNAs). Anderson et al. showed that miRNA-206 down-regulates Cx43 after birth (Anderson et al., 2006), and that this miRNA is up-regulated in turn by the myogenic transcription the factors myogenin and MyoD which promote myogenic commitment (Rao et al., 2006). In adulthood, miRNA-206 is dramatically induced in a mouse model of ALS, and delays the disease progression and promotes regeneration of neuromuscular synapses (Williams et al., 2009). However, it is known that transcription factors responsible for up-regulation of miRNA-206, MyoD and myogenin, show an increase in expression within the first week after denervation (Nikolic et al., 2010) and SCI (Dupont-Versteegden et al., 1998), which raises questions about the importance of miRNAs as regulators of the expression of Cxs during adulthood, since as mentioned above, Cxs are up-regulated under conditions of loss of nerve continuity. This situation suggests the existence of other mechanisms that regulate expression of Cxs in the adult skeletal muscle (Oyamada et al., 2005). The above-mentioned findings that the expression of Cxs in skeletal muscles is inhibited after birth, long after innervation occurs, but at a time when a marked increase in neuromotor activity is required, indicates that Cx expression levels are most likely influenced by a neuromotor activity-related mechanism.

## Mechanisms that could maintain the low expression of Cxs in adult skeletal myofibers

**Figure 1:**
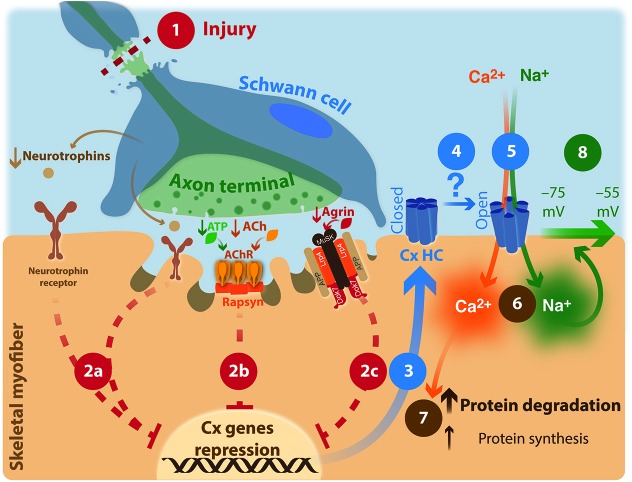
**Neuronal involvement in muscular atrophy: proposed model**. **(1)** Injury of upper- or lower motor neuron. **(2)** Repression of connexin expression by motor neuron through an unknown mechanism is interrupted. **(2a) neurotrophin signaling**; **(2b) AChR signaling**; ACh: acetylcholine; AChR: acetylcholine receptor. ATP co-released with ACh increases AChR activity. **(2c)** **agrin/Lrp4/APP/MuSK signaling**; MuSK: Muscle-Specific Kinase; Dok-7: docking protein-7; Lpr4: LDL receptor-related protein 4; APP: amyloid precursor protein. **(3)** Hemichannels formed by connexins are expressed; Cx HC: connexin hemichannel. **(4)** Increases probability of opening of Cx HC by an unknown mechanism. **(5)** Entering Ca^2+^ and Na^+^ ions through non-selective ion channels such as Cx HC **(6)**, increased cytoplasmic resting concentrations of these ions. **(7)** Protein unbalance. **(8)** Reduction in the membrane potential is generated.

The importance of Cxs throughout the life of the myofibers is rather well established, and there is considerable evidence that the neuromotor activity is related to their down-regulation in adulthood. However, the mechanism responsible for this regulation is not known. In this section we will discuss the possible mechanisms related to this issue (Figure 1).

## Acetylcholine (ACh)

The most studied function of ACh is its role in the conversion of a neuron electrical signal into a chemical signal in the NMJs to produce a mechanical response in the muscle. However, there is also another function that is less well known, but equally important regarding the development and maintenance of the NMJ.

During postsynaptic differentiation, AChR clustering is initiated by a nerve-independent mechanism (Lin et al., 2001; Yang et al., 2001). The muscle-specific receptor tyrosine kinase (MuSK) together with Wnt ligands are involved in the prepatterning of adult AChRs, organizing them into concentrated clusters (Jing et al., 2009). From the time that a motor neuron innervates a myofiber, ACh through the cyclin-dependent kinase 5 (Cdk5) pathway disperses the AChR clusters that failed to be positioned with the nerve terminal (Fu et al., 2005; Lin et al., 2005), and remains as negative signal in the formation of AChR clusters in adulthood (Misgeld et al., 2005). The Cdk5-mediated regulation of AChR localization is poorly understood; we know however that the intermediate filament protein nestin interacts with Cdk5 and is required for ACh-induced association of p35, the co-activator of Cdk5, with the muscle membrane (Yang et al., 2011).

At level of the synaptic junction, blocking the release of the synaptic vesicle with botulinum neurotoxin (Kinder et al., 2001; Jirmanova et al., 1964), or blocking of AChR with alpha bungarotoxin (Ringel et al., 1975; Shen et al., 2006), also produces a rapid onset of atrophy. Moreover, in myasthenia gravis, a pathological condition characterized by the generation of anti-AChR antibodies, also show changes in skeletal muscles similar to those induced by denervation (Berrih-Aknin and Le Panse, 2014).

On the other hand, immobilization and denervation induce *de novo* expression of neuronal nicotinic α7 AChRs (α7AChRs) in myofibers, which is Ca^2+^ permeable (Dickinson et al., 2007), and contributes to neurotransmission (Tsuneki et al., 2003; Lee et al., 2014). Thus, α7AChRs together with aforementioned non-selective channels could contribute to the increase in intracellular Ca^2+^ that occurs after denervation.

## Agrin

Agrin is a proteoglycan released by the motor nerve terminal. This protein binds MuSK and its crucial co-receptors LDL receptor-related protein 4 (Lrp4) and amyloid precursor protein (APP; Choi et al., 2013). Agrin plays a positive role in post-synaptic differentiation by inducing and maintaining AChR clustering *in vitro* and *in vivo* (Nitkin et al., 1987; McMahan, 1990; Ferns et al., 1992; Ruegg et al., 1992; DeChiara et al., 1996; Gautam et al., 1996; Glass et al., 1996; Lin et al., 2001; Yang et al., 2001). Different studies showed that the prepatterning of AChR begins before innervation (Yang et al., 2000, 2001; Lin et al., 2001). Analysis in agrin, MuSK and Lrp4 mutant mice showed that these animals die after birth due a general defect in the assembly of the postsynaptic machinery when NMJ function is required for breathing (DeChiara et al., 1996; Gautam et al., 1996; Hesser et al., 2006; Weatherbee et al., 2006). On the other hand, in absence of ACh (or synaptic transmission) and agrin, the NMJs do form normally, but mice die at birth due to respiratory failure in the absence of neuromuscular transmission (Misgeld et al., 2005). Thus, agrin appears to be responsible for stabilizing the nascent postsynaptic apparatus formed through the action of MuSK/Lrp4.

The cell signaling downstream of MuSK requires the cytoplasmic adaptor protein (Dok-7), which is essential for both MuSK-mediated prepatterning of AChRs and agrin-stimulated AChR cluster stabilization upon innervation though the mechanism is not clear (Okada et al., 2006; Inoue et al., 2009); as might be expected, mice carrying loss of function mutations of Dok-7 die shortly after birth (Beeson et al., 2006; Okada et al., 2006).

In humans, 5–10% cases of myasthenia gravis are caused by autoantibodies against MuSK, which prevent binding between MuSK and Lrp4, and inhibit agrin-stimulated MuSK phosphorylation (Huijbers et al., 2013). LDL receptor-related protein 4 has also been shown to be a target of autoantibodies in some MG patients; being a new diagnostic marker for this disease (Pevzner et al., 2012).

## ATP

ATP is recognized as an important signaling molecule that mediates diverse biological processes. In skeletal muscle, ATP is released at the NMJ by synaptic vesicles and by myofibers and has a postulated role in various regulatory processes including cell proliferation, differentiation, and muscle contraction.

Synaptic vesicles isolated from vertebrates contain ACh and ATP at a ratio of ~10:1 (Dowdall et al., 1974; Zimmermann and Denston, 1976; Volknandt and Zimmermann, 1986). An ADP/ATP translocase enables the synaptic vesicle to accumulate ATP (Luqmani, 1981; Lee and Witzemann, 1983; Stadler and Fenwick, 1983); inside the vesicle, ATP is not, however complexed with ACh (Kobos and Rechnitz, 1976). ATP is released with ACh at NMJ in a pulsatile way in response to nerve impulses (Silinsky, 1975; Redman and Silinsky, 1994; Silinsky and Redman, 1996; Vizi et al., 2000; Santos et al., 2003). The significance of this pulsatile release is not clear. In development, ATP bound to P2X receptors is equally effective with ACh acting through nicotinic receptors in calcium mobilization (Kolb and Wakelam, 1983; Häggblad and Heilbronn, 1988). In adults, the co-transmitter role of ATP is less prominent than during development. Adenosine generated by the hydrolysis of ATP was proposed as physiological mediator of prejunctional neuromuscular depression (Redman and Silinsky, 1994); at postjunctional sites, extracellular ATP facilitates the action of ACh (Ribeiro, 1977), increases AChR activity (Ewald, 1976; Akasu et al., 1981; Lu and Smith, 1991), and K^+^ channel activation (Thomas and Hume, 1993), and inhibits Cl^−^ channels, by activating P2Y_1_ receptors (Voss, 2009); overall, ATP enhances neuromuscular signaling in adult skeletal muscle.

During skeletal muscle contraction, ATP is released from muscle fibers (Cunha and Sebastião, 1993; Sandonà et al., 2005; Li et al., 2008). This ATP can be released through ATP permeable channels, including Cx HC and Panx channels (Bao et al., 2004; Kang et al., 2008; Buvinic et al., 2009; Riquelme et al., 2013). As was mentioned in Section Connexins and Skeletal Muscle, Cxs are not expressed in the adult skeletal muscle. However, the Panx1 is expressed and forms Panx1 HCs in T-tubules. Thus Panx1 channels could be responsible for the release of ATP in adult skeletal muscles. This ATP is necessary for the muscle potentiation that occurs during repetitive electrical stimulation (Riquelme et al., 2013).

The accumulation of ATP outside the sarcolemma has also been shown to be necessary for the increased membrane permeability observed in muscle in pathological conditions where it activates Cx HCs and Panx1 channels as well as P2X_7_Rs leading to membrane permeabilization to ions and small molecules (Cea et al., 2013); this accumulation of extracellular ATP is likely to be facilitated after denervation of skeletal muscles or spinal cord injury by the *de novo* expression of P2X_7_Rs and Cxs (39, 43 and 45) and up-regulation of Panx1 (Cea et al., 2013) as discussed above.

### Neurotrophic factors

The neurotrophic factors are critical for the development of the nervous system (Skaper, 2012). In adulthood, there is well-established interdependence between glial cells and motor neurons (Michailov et al., 2004; Schulz et al., 2014). However, little is known about the relationship between neurons, neurotrophic factors, and trophic actions on myofibers.

Neuregulin 1 (NRG1) has been proposed as an extracellular signal that induces synapse-specific transcription, because NRG1 induces AChR transcription in cultured muscle cells (Falls, 2003). However, mice lacking NRG1 in both motor neurons and skeletal muscles, or deficient for both the NRG-1 receptors ErbB2 and ErbB4 in skeletal muscles, have morphologically normal synapses, although the amounts of AChRs and AChR mRNA at synapses are modestly reduced (Escher et al., 2005; Jaworski and Burden, 2006). A recent work elucidates that NRG1/ErbB signaling maintains the efficacy of synaptic transmission by stabilizing the NMJs via phosphorylation of α-dystrobrevin (Schmidt et al., 2011).

Ciliary neurotrophic factor (CNTF), a member of the interleukin-6 (IL-6) superfamily, induces cachectic effects (Martin et al., 1996) and several inflammatory responses on innervated skeletal muscle, including induction of fever and a hepatic acute phase protein response (Espat et al., 1996). Also, it has been postulated to CNTF acts as a neurotrophic factor that regulates the expression of its receptor (CNTFR; Helgren et al., 1994; Ip et al., 1996) and acetylcholinesterase in adult rat skeletal muscle (Boudreau-Larivière et al., 1996).

Brain-derived neurotrophic factor (BDNF) is expressed at relatively high levels during muscle development and then down regulated postnatally (Griesbeck et al., 1995; Mousavi et al., 2004). In adult rat muscle the constitutive expression of BDNF is confined to the myofibers, satellite cells, Schwann cells and endothelial cells (Liem et al., 2001), and is up-regulated in muscles as response to acute or repeated exercise (Cuppini et al., 2007; Matthews et al., 2009), but its possible effects on myofibers are unknown.

In co-cultures of dissociated DRG neurons and skeletal myofibers nerve growth factor (NGF) and neurotrophin 3 (NT-3) increase the levels of messenger RNAs (mRNAs) of preprotachykinin (PPT), calcitonin-gene related peptide (CGRP), neurofilament 200 (NF-200), and microtubule associated protein 2 (MAP-2; Zhang et al., 2012), suggesting trophic effects although one could not assess how these growth factors altered neuromuscular function. Due to the limited information available, more studies are needed to elucidate the true importance of neurotrophic factors in the maintenance of muscle characteristics.

## Concluding remarks

Electrical and metabolic coupling mediated by Cx-based gap junctions are characteristics of smooth and cardiac muscles, which must achieve coordinated contraction of large groups of myocytes. The expression of Cx proteins is required for the formation of gap junction channels that play critical roles in coordinating diverse normal tissue functions in smooth and cardiac muscle, including propagation of cardiac action potentials and smooth muscle slow contraction waves. By contrast, skeletal muscles are characterized by precise and rapid contraction responses of single fibers or groups of fibers innervated by a common nerve fiber (motor units) that must be activated independently of muscle fibers in other motor units. This feature is accomplished by the direct command of the nervous system through nerve fibers with similar conduction velocity that innervate individual motor units with similar electrical threshold. Thus, electrical coupling of muscle fibers through gap junctions appears to be unnecessary for rapid and coordinated contraction of skeletal muscle fibers. Notably, neuromuscular activation represses the expression or translation of several non-selective ion channels during development.

Neuromuscular activation represses the expression or translation of several non-selective ion channels including HCs formed by Cxs 39, 43 or 45, P2X_7_R, TRPV2 channel and alpha-7 nicotinic receptor in skeletal myofibers (Cea et al., 2013; Lee et al., 2014). However, disruption of neural continuity at any level between upper motor neuron and motor end plate elevates the membrane incorporation of those gene products, with major adverse effects on myofiber biology. Moreover, the membrane expression of Panx1 channels is up-regulated. The sarcolemmal incorporation of these protein subunits results in the cell surface expression of non-selective ion channels, all permeable to monovalent cations and Ca^2+^ and some of them are also permeable to small molecules (e.g., Cx HCs). Therefore, all of them could contribute to different extents to reducing the resting membrane potential of denervated myofibers as well as to the activation of intracellular metabolic response activated by free cytoplasmic Ca^2+^, including protein degradation. The overlapping features of non-selective ion channels expressed in denervated muscle might be taken as evidence that their expression is controlled by a common mechanism (e.g., the same transcription factor). Thus, a critical issue to be unraveled in the future is the identification of the signal transduction mechanism activated at NMJs/motor end plates that repress the expression of all these non-selective ion channels. So far, it is known that early electrical stimulation of muscles under disuse due to denervation does not prevent the reduction in resting membrane potential and thus is insufficient to maintain the homeostasis of the sarcolemma. Likewise, the presence of an intact lower motor neuron and NMJ does not appear sufficient to prevent incorporation of these HCs and channels into the sarcolemma. Contact between the nerve terminal and motor end plate allows interaction of a series of molecules released by axon terminals and glial cells and their receptors present in myofibers. The main molecules are: ACh that is responsible for the end-plate potential and dispersal of Ach receptor clusters, ATP which is involved in muscle potentiation, agrin that acts as positive signal in the clustering of AChRs, and neurotrophins, whose effect on adult muscle fibers is poorly understood (Figure 1). Therefore, more studies are needed to elucidate the role of these substances in repressing incorporation of the above channels and HCs into the sarcolemma of skeletal muscle. miRNAs are recognized as regulators of diverse gene networks and pathways and bind to their target mRNAs, causing mRNA degradation or preventing protein translation. However, miRNA expression levels do not fully explain changes in myofiber expression levels of HCs and channels, and further studies are required to identify the role of miRNA, and to identify alternative mechanisms that determine sarcolemmal expression levels of them.

The discovery of the humoral factor that prevents the expression of protein subunits that form non-selective ion channels in denervated muscles might unveil a valuable molecular target to design a rational therapeutic to prevent degeneration of denervated myofibers that might also be useful to treat diverse myopathies with compromised NMJs.

## Conflict of interest statement

The authors declare that the research was conducted in the absence of any commercial or financial relationships that could be construed as a potential conflict of interest.

## References

[B1] Adami N. Kern H. Mayr W. Carraro U. Biral D. Sandra Z. (2007). Permanent denervation of rat Tibialis Anterior after bilateral sciatectomy: determination of chronaxie by surface electrode stimulation during progression of atrophy up to one year. Basic Appl. Myol. 17, 237–243.

[B172] Adams L. Goldman D. (1998). Role for calcium from the sarcoplasmic reticulum in coupling muscle activity to nicotinic acetylcholine receptor gene expression in rat. J. Neurobiol. 35, 245–257. 10.1002/(SICI)1097-4695(19980605)35:3%3C245::AID-NEU2%3E3.0.CO;2-Z 9622008

[B2] Akasu T. Hirai K. Koketsu K. (1981). Increase of acetylcholine-receptor sensitivity by adenosine triphosphate: a novel action of ATP on ACh-sensitivity. Br. J. Pharmacol. 74, 505–507. 10.1111/j.1476-5381.1981.tb09997.x 7317696 PMC2071722

[B164] Albuquerque E. X. Thesleff S. (1968). A comparative study of membrane properties of innervated and chronically denervated fast and slow skeletal muscles of the rat. Acta Physiol. Scand. 73, 471–480. 10.1111/j.1365-201x.1968.tb10886.x 5708174

[B3] Anderson C. Catoe H. Werner R. (2006). MIR-206 regulates connexin43 expression during skeletal muscle development. Nucleic Acids Res. 34, 5863–5871. 10.1093/nar/gkl743 17062625 PMC1635318

[B4] Araya R. Eckardt D. Riquelme M. A. Willecke K. Sáez J. C. (2003). Presence and importance of connexin43 during myogenesis. Cell Commun. Adhes. 10, 451–456. 10.1080/cac.10.4-6.451.456 14681056

[B5] Araya R. Riquelme M. A. Brandan E. Sáez J. C. (2004). The formation of skeletal muscle myotubes requires functional membrane receptors activated by extracellular ATP. Brain Res. Brain Res. Rev. 47, 174–188. 10.1016/j.brainresrev.2004.06.003 15572171

[B6] Armstrong D. L. Turin L. Warner A. E. (1983). Muscle activity and the loss of electrical coupling between striated muscle cells in Xenopus embryos. J. Neurosci. 3, 1414–1421. 6306176 10.1523/JNEUROSCI.03-07-01414.1983 PMC6564429

[B7] Award E. A. Swaiman K. F. Kottke F. J. (1965). Changes in the structure, innervation, electromyographic patterns and enzymes of skeletal muscle resulting from experimental treatment with triamcinolone. Arch. Phys. Med. Rehabil. 46, 297–306. 14280219

[B174] Bain J. R. Veltri K. L. Chamberlain D. Fahnestock M. (2001). Improved functional recovery of denervated skeletal muscle after temporary sensory nerve innervation. Neuroscience 103, 503–510. 10.1016/s0306-4522(00)00577-7 11246164

[B8] Baldi J. C. Jackson R. D. Moraille R. Mysiw W. J. (1998). Muscle atrophy is prevented in patients with acute spinal cord injury using functional electrical stimulation. Spinal Cord 36, 463–469. 10.1038/sj.sc.3100679 9670381

[B9] Baldwin K. M. Haddad F. Pandorf C. E. Roy R. R. Edgerton V. R. (2013). Alterations in muscle mass and contractile phenotype in response to unloading models: role of transcriptional/pretranslational mechanisms. Front. Physiol. 4:284. 10.3389/fphys.2013.00284 24130531 PMC3795307

[B10] Bao L. Locovei S. Dahl G. (2004). Pannexin membrane channels are mechanosensitive conduits for ATP. FEBS Lett. 572, 65–68. 10.1016/j.febslet.2004.07.009 15304325

[B11] Beeson D. Higuchi O. Palace J. Cossins J. Spearman H. Maxwell S.. (2006). Dok-7 mutations underlie a neuromuscular junction synaptopathy. Science 313, 1975–1978. 10.1126/science.1130837 16917026

[B12] Belluardo N. Trovato-Salinaro A. Mudò G. Condorelli D. F. (2005). Expression of the rat connexin 39 (rCx39) gene in myoblasts and myotubes in developing and regenerating skeletal muscles: an in situ hybridization study. Cell Tissue Res. 320, 299–310. 10.1007/s00441-005-1087-7 15778849

[B13] Berrih-Aknin S. Le Panse R. (2014). Myasthenia gravis: a comprehensive review of immune dysregulation and etiological mechanisms. J. Autoimmun. 52, 90–100. 10.1016/j.jaut.2013.12.011 24389034

[B14] Birks R. Katz B. Miledi R. (1960). Physiological and structural changes at the amphibian myoneural junction, in the course of nerve degeneration. J. Physiol. 150, 145–168. 13800902 10.1113/jphysiol.1960.sp006379 PMC1363153

[B15] Bjugn R. Nyengaard J. R. Rosland J. H. (1997). Spinal cord transection–no loss of distal ventral horn neurons. Exp. Neurol. 148, 179–186. 10.1006/exnr.1997.6610 9398460

[B16] Boudreau-Larivière C. Sveistrup H. Parry D. J. Jasmin B. J. (1996). Ciliary neurotrophic factor: regulation of acetylcholinesterase in skeletal muscle and distribution of messenger RNA encoding its receptor in synaptic versus extrasynaptic compartments. Neuroscience 73, 613–622. 10.1016/0306-4522(96)00033-4 8783275

[B17] Bruusgaard J. C. Gundersen K. (2008). In vivo time-lapse microscopy reveals no loss of murine myonuclei during weeks of muscle atrophy. J. Clin. Invest. 118, 1450–1457. 10.1172/JCI34022 18317591 PMC2262032

[B175] Bruzzone S. Guida L. Zocchi E. Franco L. De Flora A. (2001). Connexin 43 hemi channels mediate Ca2+-regulated transmembrane NAD+ fluxes in intact cells. FASEB J. 15, 10–12. 10.1096/fj.00-0566fje 11099492

[B18] Burns A. S. Jawaid S. Zhong H. Yoshihara H. Bhagat S. Murray M.. (2007). Paralysis elicited by spinal cord injury evokes selective disassembly of neuromuscular synapses with and without terminal sprouting in ankle flexors of the adult rat. J. Comp. Neurol. 500, 116–133. 10.1002/cne.21143 17099885

[B19] Buvinic S. Almarza G. Bustamante M. Casas M. López J. Riquelme M.. (2009). ATP released by electrical stimuli elicits calcium transients and gene expression in skeletal muscle. J. Biol. Chem. 284, 34490–34505. 10.1074/jbc.M109.057315 19822518 PMC2787310

[B20] Castro M. J. Apple D. F. Jr. Staron R. S. Campos G. E. Dudley G. A. (1999). Influence of complete spinal cord injury on skeletal muscle within 6 mo of injury. J. Appl. Physiol. (1985) 86, 350–358. 9887150 10.1152/jappl.1999.86.1.350

[B21] Cea L. A. Cisterna B. A. Puebla C. Frank M. Figueroa X. F. Cardozo C.. (2013). De novo expression of connexin hemichannels in denervated fast skeletal muscles leads to atrophy. Proc. Natl. Acad. Sci. U S A 110, 16229–16234. 10.1073/pnas.1312331110 24043768 PMC3791696

[B22] Cherian P. P. Siller-Jackson A. J. Gu S. Wang X. Bonewald L. F. Sprague E.. (2005). Mechanical strain opens connexin 43 hemichannels in osteocytes: a novel mechanism for the release of prostaglandin. Mol. Biol Cell 16, 3100–3106. 10.1091/mbc.e04-10-0912 15843434 PMC1165395

[B23] Choi H. Y. Liu Y. Tennert C. Sugiura Y. Karakatsani A. Kroger S.. (2013). APP interacts with LRP4 and agrin to coordinate the development of the neuromuscular junction in mice. Elife 2:e00220. 10.7554/elife.00220 23986861 PMC3748711

[B24] Constantin B. Cronier L. (2000). Involvement of gap junctional communication in myogenesis. Int. Rev. Cytol. 196, 1–65. 10.1016/S0074-7696(00)96001-7 10730212

[B25] Cunha R. A. Sebastião A. M. (1993). Adenosine and adenine nucleotides are independently released from both the nerve terminals and the muscle fibres upon electrical stimulation of the innervated skeletal muscle of the frog. Pflugers Arch. 424, 503–510. 10.1007/bf00374914 8255734

[B26] Cuppini R. Sartini S. Agostini D. Guescini M. Ambrogini P. Betti M.. (2007). Bdnf expression in rat skeletal muscle after acute or repeated exercise. Arch. Ital. Biol. 145, 99–110. 17639782

[B27] DeChiara T. M. Bowen D. C. Valenzuela D. M. Simmons M. V. Poueymirou W. T. Thomas S.. (1996). The receptor tyrosine kinase MuSK is required for neuromuscular junction formation in vivo. Cell 85, 501–512. 10.1016/s0092-8674(00)81251-9 8653786

[B28] Dell D. D. (2002). Cachexia in patients with advanced cancer. Clin. J. Oncol. Nurs. 6, 235–238. 10.1188/02.CJON.235-238 12087622

[B29] Demontis F. Piccirillo R. Goldberg A. L. Perrimon N. (2013). Mechanisms of skeletal muscle aging: insights from Drosophila and mammalian models. Dis. Model. Mech. 6, 1339–1352. 10.1242/dmm.012559 24092876 PMC3820258

[B30] Dennis M. J. Ziskind-Conhaim L. Harris A. J. (1981). Development of neuromuscular junctions in rat embryos. Dev. Biol. 81, 266–279. 10.1016/0012-1606(81)90290-6 7202841

[B179] Dickinson J. A. Hanrott K. E. Mok M. H. Kew J. N. Wonnacott S. (2007). Differential coupling of alpha7 and non-alpha7 nicotinic acetylcholine receptors to calcium-induced calcium release and voltage-operated calcium channels in PC12 cells. J. Neurochem. 100, 1089–1096. 10.1111/j.1471-4159.2006.04273.x 17181555

[B31] Ditunno J. F. Little J. W. Tessler A. Burns A. S. (2004). Spinal shock revisited: a four-phase model. Spinal Cord 42, 383–395. 10.1038/sj.sc.3101603 15037862

[B32] Dowdall M. J. Boyne A. F. Whittaker V. P. (1974). Adenosine triphosphate. A constituent of cholinergic synaptic vesicles. Biochem. J. 140, 1–12. 4451548 10.1042/bj1400001 PMC1167964

[B33] Dudley-Javoroski S. Shields R. K. (2008). Muscle and bone plasticity after spinal cord injury: review of adaptations to disuse and to electrical muscle stimulation. J. Rehabil. Res. Dev. 45, 283–296. 10.1682/jrrd.2007.02.0031 18566946 PMC2744487

[B34] Dupont-Versteegden E. E. Houlé J. D. Gurley C. M. Peterson C. A. (1998). Early changes in muscle fiber size and gene expression in response to spinal cord transection and exercise. Am. J. Physiol. 275, C1124–C1133. 9755066 10.1152/ajpcell.1998.275.4.C1124

[B35] Emmanouilidou E. Melachroinou K. Roumeliotis T. Garbis S. D. Ntzouni M. Margaritis L. H.. (2010). Cell-produced alpha-synuclein is secreted in a calcium-dependent manner by exosomes and impacts neuronal survival. J. Neurosci. 30, 6838–6851. 10.1523/JNEUROSCI.5699-09.2010 20484626 PMC3842464

[B36] Escher P. Lacazette E. Courtet M. Blindenbacher A. Landmann L. Bezakova G.. (2005). Synapses form in skeletal muscles lacking neuregulin receptors. Science 308, 1920–1923. 10.1126/science.1108258 15976301

[B37] Espat N. J. Auffenberg T. Rosenberg J. J. Rogy M. Martin D. Fang C. H.. (1996). Ciliary neurotrophic factor is catabolic and shares with IL-6 the capacity to induce an acute phase response. Am. J. Physiol. 271, R185–R190. 8760219 10.1152/ajpregu.1996.271.1.R185

[B38] Ewald D. A. (1976). Potentiation of postjunctional cholinergic sensitivity of rat diaphragm muscle by high-energy-phosphate adenine nucleotides. J. Membr. Biol. 29, 47–65. 10.1007/bf01868951 185389

[B39] Eyzaguirre C. Espildora J. Luco J. V. (1952). Alterations of neuromuscular synapsis during Wallerian degeneration. Acta Physiol. Lat. Am. 2, 213–227. 13040034

[B40] Falls D. L. (2003). Neuregulins and the neuromuscular system: 10 years of answers and questions. J. Neurocytol. 32, 619–647. 10.1023/b:neur.0000020614.83883.be 15034257

[B41] Ferns M. Hoch W. Campanelli J. T. Rupp F. Hall Z. W. Scheller R. H. (1992). RNA splicing regulates agrin-mediated acetylcholine receptor clustering activity on cultured myotubes. Neuron 8, 1079–1086. 10.1016/0896-6273(92)90129-2 1319184

[B42] Fiori M. C. Figueroa V. Zoghbi M. E. Sáez J. C. Reuss L. Altenberg G. A. (2012). Permeation of calcium through purified connexin 26 hemichannels. J. Biol. Chem. 287, 40826–40834. 10.1074/jbc.M112.383281 23048025 PMC3504794

[B43] Fu A. K. Ip F. C. Fu W. Y. Cheung J. Wang J. H. Yung W. H.. (2005). Aberrant motor axon projection, acetylcholine receptor clustering and neurotransmission in cyclin-dependent kinase 5 null mice. Proc. Natl. Acad. Sci. U S A 102, 15224–15229. 10.1073/pnas.0507678102 16203963 PMC1257743

[B44] Gage P. W. Lamb G. D. Wakefield B. T. (1989). Transient and persistent sodium currents in normal and denervated mammalian skeletal muscle. J. Physiol. 418, 427–439. 2559972 10.1113/jphysiol.1989.sp017850 PMC1189981

[B45] Gautam M. Noakes P. G. Moscoso L. Rupp F. Scheller R. H. Merlie J. P.. (1996). Defective neuromuscular synaptogenesis in agrin-deficient mutant mice. Cell 85, 525–535. 10.1016/s0092-8674(00)81253-2 8653788

[B46] Glass D. J. (2003). Molecular mechanisms modulating muscle mass. Trends Mol. Med. 9, 344–350. 10.1016/s1471-4914(03)00138-2 12928036

[B47] Glass D. J. Bowen D. C. Stitt T. N. Radziejewski C. Bruno J. Ryan T. E.. (1996). Agrin acts via a MuSK receptor complex. Cell 85, 513–523. 10.1016/s0092-8674(00)81252-0 8653787

[B177] Gossman D. G. Zhao H. B. (2008). Hemichannel-mediated inositol 1,4,5-trisphosphate (IP3) release in the cochlea: a novel mechanism of IP3 intercellular signaling. Cell Commun. Adhes. 15, 305–315. 10.1080/15419060802357217 18979296 PMC5543712

[B48] Griesbeck O. Parsadanian A. S. Sendtner M. Thoenen H. (1995). Expression of neurotrophins in skeletal muscle: quantitative comparison and significance for motoneuron survival and maintenance of function. J. Neurosci. Res. 42, 21–33. 10.1002/jnr.490420104 8531223

[B161] Gutmann E. Vodicka Z. Zelená J. (1955). Veranderungen in quergestreiften Muskel bei Durchtrennung in Abhangigkeit von der Lange des peripheren Stumpfes. Physiologia Bohemoslov. 4, 200–204.

[B170] Häggblad J. Heilbronn E. (1988). P2-purinoceptor-stimulated phosphoinositide turnover in chick myotubes. Calcium mobilization and the role of guanyl nucleotide-binding proteins. FEBS Lett. 235, 133–136. 10.1016/0014-5793(88)81248-1 2841152

[B49] Harris R. L. W. Bobet J. Sanelli L. Bennett D. J. (2006). Tail muscles become slow but fatigable in chronic sacral spinal rats with spasticity. J. Neurophysiol. 95, 1124–1133. 10.1152/jn.00456.2005 16282205 PMC5726403

[B165] Harris J. B. Thesleff S. (1971). Studies on tetrodotoxin resistant action potentials in denervated skeletal muscle. Acta Physiol. Scand. 83, 382–388. 10.1111/j.1748-1716.1971.tb05091.x 5134176

[B50] Helgren M. E. Squinto S. P. Davis H. L. Parry D. J. Boulton T. G. Heck C. S.. (1994). Trophic effect of ciliary neurotrophic factor on denervated skeletal muscle. Cell 76, 493–504. 10.1016/0092-8674(94)90113-9 8313470

[B51] Hesser B. A. Henschel O. Witzemann V. (2006). Synapse disassembly and formation of new synapses in postnatal muscle upon conditional inactivation of MuSK. Mol. Cell. Neurosci. 31, 470–480. 10.1016/j.mcn.2005.10.020 16337809

[B52] Hník P. Kkorpil V. Vyklický L. (1962). “Diagnosis and therapy of denervation muscle atrophy,” in The Denervated Muscle, ed Gutmann E. (Prague: Publishing House of the Czechoslovak Academy of Science), 433–466.

[B53] Hník P. Skorpil V. (1962). “Fibrillation activity in denervated muscle,” in The Denervated Muscle, ed Gutmann E. (Prague: Publishing House of the Czechoslovak Academy of Sciences), 136–150.

[B54] Huijbers M. G. Zhang W. Klooster R. Niks E. H. Friese M. B. Straasheijm K. R.. (2013). MuSK IgG4 autoantibodies cause myasthenia gravis by inhibiting binding between MuSK and Lrp4. Proc. Natl. Acad. Sci. U S A 110, 20783–20788. 10.1073/pnas.1313944110 24297891 PMC3870730

[B55] Hyatt J. P. Roy R. R. Baldwin K. M. Edgerton V. R. (2003). Nerve activity-independent regulation of skeletal muscle atrophy: role of MyoD and myogenin in satellite cells and myonuclei. Am. J. Physiol. Cell Physiol. 285, C1161–C1173. 10.1152/ajpcell.00128.2003 12839833

[B56] Inoue A. Setoguchi K. Matsubara Y. Okada K. Sato N. Iwakura Y.. (2009). Dok-7 activates the muscle receptor kinase MuSK and shapes synapse formation. Sci. Signal. 2:ra7. 10.1126/scisignal.2000113 19244212

[B57] Ip F. C. Fu A. K. Tsim K. W. Ip N. Y. (1996). Differential expression of ciliary neurotrophic factor receptor in skeletal muscle of chick and rat after nerve injury. J. Neurochem. 67, 1607–1612. 10.1046/j.1471-4159.1996.67041607.x 8858945

[B58] Ishii W. Matsuda M. Okamoto N. Shimojima Y. Yazaki M. Motomura M.. (2005). Myasthenia gravis with anti-MuSK antibody, showing progressive muscular atrophy without blepharoptosis. Intern. Med. 44, 671–672. 10.2169/internalmedicine.44.671 16020904

[B59] Jackman R. W. Cornwell E. W. Wu C. L. Kandarian S. C. (2013). Nuclear factor-kappaB signalling and transcriptional regulation in skeletal muscle atrophy. Exp. Physiol. 98, 19–24. 10.1113/expphysiol.2011.063321 22848079 PMC3505235

[B60] Jaworski A. Burden S. J. (2006). Neuromuscular synapse formation in mice lacking motor neuron- and skeletal muscle-derived Neuregulin-1. J. Neurosci. 26, 655–661. 10.1523/jneurosci.4506-05.2006 16407563 PMC6674415

[B181] Jing L. Lefebvre J. L. Gordon L. R. Granato M. (2009). Wnt signals organize synaptic prepattern and axon guidance through the zebrafish unplugged/MuSK receptor. Neuron 61, 721–733. 10.1016/j.neuron.2008.12.025 19285469 PMC2671566

[B163] Jirmanova I. Sobotkova M. Thesleff S. Zelena J. (1964). Atrophy in skeletal muscles poisoned with botulinum toxin. Physiol. Bohemoslov. 13, 467–472. 14212482

[B61] Jones R. Vrbová G. (1970). Effect of muscle activity on denervation hypersensitivity. J. Physiol. 210, 144P–145P. 5503488

[B62] Kaelan C. Jacobsen P. F. Kakulas B. A. (1988). An investigation of possible transynaptic neuronal degeneration in human spinal cord injury. J. Neurol. Sci. 86, 231–237. 10.1016/0022-510x(88)90101-3 3221242

[B63] Kalderon N. Epstein M. L. Gilula N. B. (1977). Cell-to-cell communication and myogenesis. J. Cell Biol. 75, 788–806. 10.1083/jcb.75.3.788 925080 PMC2111599

[B64] Kang J. Kang N. Lovatt D. Torres A. Zhao Z. Lin J.. (2008). Connexin 43 hemichannels are permeable to ATP. J. Neurosci. 28, 4702–4711. 10.1523/JNEUROSCI.5048-07.2008 18448647 PMC3638995

[B65] Keesey J. C. (2004). Clinical evaluation and management of myasthenia gravis. Muscle Nerve 29, 484–505. 10.1002/mus.20030 15052614

[B66] Kim S. J. Roy R. R. Kim J. A. Zhong H. Haddad F. Baldwin K. M.. (2008). Gene expression during inactivity-induced muscle atrophy: effects of brief bouts of a forceful contraction countermeasure. J. Appl. Physiol. (1985) 105, 1246–1254. 10.1152/japplphysiol.90668.2008 18653749 PMC2576041

[B67] Kinder F. R. Jr. Versace R. W. Bair K. W. Bontempo J. M. Cesarz D. Chen S.. (2001). Synthesis and antitumor activity of ester-modified analogues of bengamide B. J. Med. Chem. 44, 3692–3699. 10.1021/jm010188c 11606134

[B68] Kobos R. K. Rechnitz G. A. (1976). Acetylcholine-ATP binding by direct membrane electrode measurement. Biochem. Biophys. Res. Commun. 71, 762–767. 10.1016/0006-291x(76)90896-2 9084

[B69] Kolb H. A. Wakelam M. J. (1983). Transmitter-like action of ATP on patched membranes of cultured myoblasts and myotubes. Nature 303, 621–623. 10.1038/303621a0 6304532

[B70] Kotsias B. A. Venosa R. A. (2001). Sodium influx during action potential in innervated and denervated rat skeletal muscles. Muscle Nerve 24, 1026–1033. 10.1002/mus.1106 11439377

[B71] Lee D. A. Witzemann V. (1983). Photoaffinity labeling of a synaptic vesicle specific nucleotide transport system from Torpedo marmorata. Biochemistry 22, 6123–6130. 10.1021/bi00295a013 6689272

[B72] Lee S. Yang H. S. Sasakawa T. Khan M. A. Khatri A. Kaneki M.. (2014). Immobilization with atrophy induces de novo expression of neuronal nicotinic alpha7 acetylcholine receptors in muscle contributing to neurotransmission. Anesthesiology 120, 76–85. 10.1097/ALN.0000000000000025 24126263 PMC3910258

[B73] Li C. L. (1960). Mechanism of fibrillation potentials in denervated mammalian skeletal muscle. Science 132, 1889–1890. 10.1126/science.132.3443.1889 13761816

[B75] Li J. Gao Z. Kehoe V. Xing J. King N. Sinoway L. (2008). Interstitial adenosine triphosphate modulates muscle afferent nerve-mediated pressor reflex. Muscle Nerve 38, 972–977. 10.1002/mus.21014 18570238 PMC3600608

[B74] Li F. Sugishita K. Su Z. Ueda I. Barry W. H. (2001). Activation of connexin-43 hemichannels can elevate [Ca(2+)]i and [Na(+)]i in rabbit ventricular myocytes during metabolic inhibition. J. Mol. Cell. Cardiol. 33, 2145–2155. 10.1006/jmcc.2001.1477 11735261

[B76] Liem R. S. Brouwer N. Copray J. C. (2001). Ultrastructural localisation of intramuscular expression of BDNF mRNA by silver-gold intensified non-radioactive in situ hybridisation. Histochem. Cell Biol. 116, 545–551. 10.1007/s00418-001-0349-z 11810196

[B77] Lin W. Burgess R. W. Dominguez B. Pfaff S. L. Sanes J. R. Lee K. F. (2001). Distinct roles of nerve and muscle in postsynaptic differentiation of the neuromuscular synapse. Nature 410, 1057–1064. 10.1038/35074025 11323662

[B78] Lin W. Dominguez B. Yang J. Aryal P. Brandon E. P. Gage F. H.. (2005). Neurotransmitter acetylcholine negatively regulates neuromuscular synapse formation by a Cdk5-dependent mechanism. Neuron 46, 569–579. 10.1016/j.neuron.2005.04.002 15944126

[B79] Ling Y. Appelt D. Kelly A. M. Franzini-Armstrong C. (1992). Differences in the histogenesis of EDL and diaphragm in rat. Dev. Dyn. 193, 359–369. 10.1002/aja.1001930409 1511175

[B166] Lomo T. Rosenthal J. (1972). Control of ACh sensitivity by muscle activity in the rat. J. Physiol. 221, 493–513. 4336524 10.1113/jphysiol.1972.sp009764 PMC1331346

[B80] Lomo T. Westgaard R. H. (1975). Further studies on the control of ACh sensitivity by muscle activity in the rat. J. Physiol. 252, 603–626. 1206569 10.1113/jphysiol.1975.sp011161 PMC1348486

[B81] Lu Z. Smith D. O. (1991). Adenosine 5^′^-triphosphate increases acetylcholine channel opening frequency in rat skeletal muscle. J. Physiol. 436, 45–56. 2061841 10.1113/jphysiol.1991.sp018538 PMC1181493

[B82] Luco J. V. Eyzaguirre C. (1955). Fibrillation and hypersensitivity to ACh in denervated muscle: effect of length of degenerating nerve fibers. J. Neurophysiol. 18, 65–73. 13222158 10.1152/jn.1955.18.1.65

[B83] Luqmani Y. A. (1981). Nucleotide uptake by isolated cholinergic synaptic vesicles: evidence for a carrier of adenosine 5′-triphosphate. Neuroscience 6, 1011–1021. 10.1016/0306-4522(81)90067-1 7279210

[B84] Ma J. Shen J. Garrett J. P. Lee C. A. Li Z. Elsaidi G. A.. (2007). Gene expression of myogenic regulatory factors, nicotinic acetylcholine receptor subunits and GAP-43 in skeletal muscle following denervation in a rat model. J. Orthop. Res. 25, 1498–1505. 10.1002/jor.20414 17568415

[B85] Maeda K. Ueda M. Ohtaka H. Koyama Y. Ohgami M. Miyazaki H. (1987). A massive dose of vincristine. Jpn. J. Clin. Oncol. 17, 247–253. 3669366

[B86] Martin D. Merkel E. Tucker K. K. McManaman J. L. Albert D. Relton J.. (1996). Cachectic effect of ciliary neurotrophic factor on innervated skeletal muscle. Am. J. Physiol. 271, R1422–R1428. 8945982 10.1152/ajpregu.1996.271.5.R1422

[B87] Matthews V. B. Aström M. B. Chan M. H. Bruce C. R. Krabbe K. S. Prelovsek O.. (2009). Brain-derived neurotrophic factor is produced by skeletal muscle cells in response to contraction and enhances fat oxidation via activation of AMP-activated protein kinase. Diabetologia 52, 1409–1418. 10.1007/s00125-009-1364-1 19387610

[B88] Maynard F. M. Karunas R. S. Waring W. P. 3rd (1990). Epidemiology of spasticity following traumatic spinal cord injury. Arch. Phys. Med. Rehabil. 71, 566–569. 2369291

[B89] McMahan U. J. (1990). The agrin hypothesis. Cold Spring Harb. Symp. Quant. Biol. 55, 407–418. 10.1101/sqb.1990.055.01.041 1966767

[B90] Michailov G. V. Sereda M. W. Brinkmann B. G. Fischer T. M. Haug B. Birchmeier C.. (2004). Axonal neuregulin-1 regulates myelin sheath thickness. Science 304, 700–703. 10.1126/science.1095862 15044753

[B91] Miledi R. Slater C. R. (1970). On the degeneration of rat neuromuscular junctions after nerve section. J. Physiol. 207, 507–528. 5499034 10.1113/jphysiol.1970.sp009076 PMC1348721

[B92] Misgeld T. Kummer T. T. Lichtman J. W. Sanes J. R. (2005). Agrin promotes synaptic differentiation by counteracting an inhibitory effect of neurotransmitter. Proc. Natl. Acad. Sci. U S A 102, 11088–11093. 10.1073/pnas.0504806102 16043708 PMC1182450

[B93] Morley J. E. (2012). Undernutrition in older adults. Fam. Pract. 29(Suppl. 1), i89–i93. 10.1093/fampra/cmr054 22399563

[B94] Mousavi K. Parry D. J. Jasmin B. J. (2004). BDNF rescues myosin heavy chain IIB muscle fibers after neonatal nerve injury. Am. J. Physiol. Cell Physiol. 287, C22–C29. 10.1152/ajpcell.00583.2003 14973145

[B95] Nikolic M. Bajek S. Bobinac D. Soic Vranic T. Starcevoc Klasan G. Arbanas J. (2010). Expression of myogenic regulatory factors in rat skeletal muscles after denervation. Periodicum Biologorum 112, 83–88.

[B96] Nitkin R. M. Smith M. A. Magill C. Fallon J. R. Yao Y. M. Wallace B. G.. (1987). Identification of agrin, a synaptic organizing protein from Torpedo electric organ. J. Cell Biol. 105, 2471–2478. 10.1083/jcb.105.6.2471 2826489 PMC2114709

[B97] Okada K. Inoue A. Okada M. Murata Y. Kakuta S. Jigami T.. (2006). The muscle protein Dok-7 is essential for neuromuscular synaptogenesis. Science 312, 1802–1805. 10.1126/science.1127142 16794080

[B98] Ollivier-Lanvin K. Lemay M. A. Tessler A. Burns A. S. (2009). Neuromuscular transmission failure and muscle fatigue in ankle muscles of the adult rat after spinal cord injury. J. Appl. Physiol. (1985) 107, 1190–1194. 10.1152/japplphysiol.00282.2009 19644032 PMC2763831

[B99] Oyamada M. Oyamada Y. Takamatsu T. (2005). Regulation of connexin expression. Biochim. Biophys. Acta 1719, 6–23. 10.1016/j.bbamem.2005.11.002 16359940

[B100] Pellegrino C. Franzini C. (1963). An electron microscope study of denervation atrophy in red and white skeletal muscle fibers. J. Cell Biol. 17, 327–349. 10.1083/jcb.17.2.327 19866627 PMC2106209

[B171] Péréon Y. Sorrentino V. Dettbarn C. Noireaud J. Palade P. (1997). Dihydropyridine receptor and ryanodine receptor gene expression in long-term denervated rat muscles. Biochem. Biophys. Res. Commun. 240, 612–617. 10.1006/bbrc.1997.7712 9398613

[B101] Pevzner A. Schoser B. Peters K. Cosma N. C. Karakatsani A. Schalke B.. (2012). Anti-LRP4 autoantibodies in AChR- and MuSK-antibody-negative myasthenia gravis. J. Neurol. 259, 427–435. 10.1007/s00415-011-6194-7 21814823

[B102] Picken J. R. Kirby A. C. (1976). Denervated frog skeletal muscle: calcium content and kinetics of exchange. Exp. Neurol. 53, 64–70. 10.1016/0014-4886(76)90281-8 964345

[B103] Proulx A. Merrifield P. A. Naus C. C. (1997). Blocking gap junctional intercellular communication in myoblasts inhibits myogenin and MRF4 expression. Dev. Genet. 20, 133–144. 10.1002/(sici)1520-6408(1997)20:2<133::aid-dvg6>3.0.co;2-8 9144924

[B167] Purves D. Sakmann B. (1974a). The effect of contractile activity on fibrillation and extrajunctional acetylcholine-sensitivity in rat muscle maintained in organ culture. J. Physiol. 237, 157–182. 4856656 10.1113/jphysiol.1974.sp010475 PMC1350874

[B104] Purves D. Sakmann B. (1974b). Membrane properties underlying spontaneous activity of denervated muscle fibre. J. Physiol. 239, 125–153. 4853156 10.1113/jphysiol.1974.sp010559 PMC1330941

[B105] Qin W. Bauman W. A. Cardozo C. (2010). Bone and muscle loss after spinal cord injury: organ interactions. Ann. N Y Acad. Sci. 1211, 66–84. 10.1111/j.1749-6632.2010.05806.x 21062296

[B106] Rahimov F. Kunkel L. M. (2013). The cell biology of disease: cellular and molecular mechanisms underlying muscular dystrophy. J. Cell Biol. 201, 499–510. 10.1083/jcb.201212142 23671309 PMC3653356

[B107] Rana S. Dringen R. (2007). Gap junction hemichannel-mediated release of glutathione from cultured rat astrocytes. Neurosci. Lett. 415, 45–48. 10.1016/j.neulet.2006.12.043 17222973

[B178] Rao P. K. Kumar R. M. Farkhondeh M. Baskerville S. Lodish H. F. (2006). Myogenic factors that regulate expression of muscle-specific microRNAs. Proc. Natl. Acad. Sci. U S A 103, 8721–8726. 10.1073/pnas.0602831103 16731620 PMC1482645

[B108] Redman R. S. Silinsky E. M. (1994). ATP released together with acetylcholine as the mediator of neuromuscular depression at frog motor nerve endings. J. Physiol. 477, 117–127. 8071878 10.1113/jphysiol.1994.sp020176 PMC1155579

[B109] Retamal M. A. Froger N. Palacios-Prado N. Ezan P. Sáez P. J. Sáez J. C.. (2007). Cx43 hemichannels and gap junction channels in astrocytes are regulated oppositely by proinflammatory cytokines released from activated microglia. J. Neurosci. 27, 13781–13792. 10.1523/jneurosci.2042-07.2007 18077690 PMC6673621

[B110] Ribeiro J. A. (1977). Potentiation of postjunctional cholinergic sensitivity of rat diaphragm muscle by high-energy-phosphate adenine nucleotides. J. Membr. Biol. 33, 401–402. 10.1007/bf01869526 864696

[B168] Ringel S. P. Bender A. N. Engel W. K. Daniels M. P. Vogel Z. (1975). A sequential study of denervation - ultrastructural immunoperoxidase localization of alpha-bungarotoxin. Trans. Am. Neurol. Assoc. 100, 52–56. 775726

[B111] Riquelme M. A. Cea L. A. Vega J. L. Boric M. P. Monyer H. Bennett M. V.. (2013). The ATP required for potentiation of skeletal muscle contraction is released via pannexin hemichannels. Neuropharmacology 75, 594–603. 10.1016/j.neuropharm.2013.03.022 23583931

[B112] Rochester L. Barron M. J. Chandler C. S. Sutton R. A. Miller S. Johnson M. A. (1995). Influence of electrical stimulation of the tibialis anterior muscle in paraplegic subjects. 2. Morphological and histochemical properties. Paraplegia 33, 514–522. 10.1038/sc.1995.112 8524604

[B160] Rosenblueth A. Luco J. V. (1937). A study of denervated mammalian skeletal muscle. Am. J. Physiol. 120, 781–797.

[B113] Rüegg M. A. Glass D. J. (2011). Molecular mechanisms and treatment options for muscle wasting diseases. Annu. Rev. Pharmacol. Toxicol. 51, 373–395. 10.1146/annurev-pharmtox-010510-100537 20936944

[B114] Ruegg M. A. Tsim K. W. Horton S. E. Kröger S. Escher G. Gensch E. M.. (1992). The agrin gene codes for a family of basal lamina proteins that differ in function and distribution. Neuron 8, 691–699. 10.1016/0896-6273(92)90090-z 1314621

[B115] Sáez J. C. Retamal M. A. Basilio D. Bukauskas F. F. Bennett M. V. (2005). Connexin-based gap junction hemichannels: gating mechanisms. Biochim. Biophys. Acta 1711, 215–224. 10.1016/j.bbamem.2005.01.014 15955306 PMC3617572

[B116] Salmons S. Ashley Z. Sutherland H. Russold M. F. Li F. Jarvis J. C. (2005). Functional electrical stimulation of denervated muscles: basic issues. Artif. Organs 29, 199–202. 10.1111/j.1525-1594.2005.29034.x 15725216

[B117] Salzer J. L. Bunge R. P. (1980). Studies of Schwann cell proliferation. I. An analysis in tissue culture of proliferation during development, Wallerian degeneration and direct injury. J. Cell Biol. 84, 739–752. 10.1083/jcb.84.3.739 6244318 PMC2110577

[B118] Sánchez H. A. Orellana J. A. Verselis V. K. Sáez J. C. (2009). Metabolic inhibition increases activity of connexin-32 hemichannels permeable to Ca2+ in transfected HeLa cells. Am. J. Physiol. Cell Physiol. 297, C665–C678. 10.1152/ajpcell.00200.2009 19587218 PMC2740400

[B119] Sandonà D. Danieli-Betto D. Germinario E. Biral D. Martinello T. Lioy A.. (2005). The T-tubule membrane ATP-operated P2X4 receptor influences contractility of skeletal muscle. FASEB J. 19, 1184–1186. 10.1096/fj.04-3333fje 15857823

[B120] Santos D. A. Salgado A. I. Cunha R. A. (2003). ATP is released from nerve terminals and from activated muscle fibres on stimulation of the rat phrenic nerve. Neurosci. Lett. 338, 225–228. 10.1016/s0304-3940(02)01419-2 12581837

[B121] Schalper K. A. Sánchez H. A. Lee S. C. Altenberg G. A. Nathanson M. H. Sáez J. C. (2010). Connexin 43 hemichannels mediate the Ca2+ influx induced by extracellular alkalinization. Am. J. Physiol. Cell Physiol. 299, C1504–C1515. 10.1152/ajpcell.00015.2010 20881238 PMC3774097

[B122] Schmidt N. Akaaboune M. Gajendran N. Martinez-Pena y Valenzuela I. Wakefield S. Thurnheer R.. (2011). Neuregulin/ErbB regulate neuromuscular junction development by phosphorylation of alpha-dystrobrevin. J. Cell Biol. 195, 1171–1184. 10.1083/jcb.201107083 22184199 PMC3246897

[B123] Schulz A. Kyselyova A. Baader S. L. Jung M. J. Zoch A. Mautner V. F.. (2014). Neuronal merlin influences ERBB2 receptor expression on Schwann cells through neuregulin 1 type III signalling. Brain 137, 420–432. 10.1093/brain/awt327 24309211 PMC3914471

[B124] Scremin A. M. Kurta L. Gentili A. Wiseman B. Perell K. Kunkel C.. (1999). Increasing muscle mass in spinal cord injured persons with a functional electrical stimulation exercise program. Arch. Phys. Med. Rehabil. 80, 1531–1536. 10.1016/s0003-9993(99)90326-x 10597802

[B125] Sekiguchi K. Kanda F. Mitsui S. Kohara N. Chihara K. (2012). Fibrillation potentials of denervated rat skeletal muscle are associated with expression of cardiac-type voltage-gated sodium channel isoform Nav1.5. Clin. Neurophysiol. 123, 1650–1655. 10.1016/j.clinph.2012.01.002 22336133

[B126] Shen J. Ma J. Lee C. Smith B. P. Smith T. L. Tan K. H.. (2006). How muscles recover from paresis and atrophy after intramuscular injection of botulinum toxin A: study in juvenile rats. J. Orthop. Res. 24, 1128–1135. 10.1002/jor.20131 16602109

[B180] Shields R. K. Dudley-Javoroski S. (2007). Musculoskeletal adaptations in chronic spinal cord injury: effects of long-term soleus electrical stimulation training. Neurorehabil. Neural Repair 21, 169–179. 10.1177/1545968306293447 17312092 PMC3270314

[B127] Silinsky E. M. (1975). On the association between transmitter secretion and the release of adenine nucleotides from mammalian motor nerve terminals. J. Physiol. 247, 145–162. 166162 10.1113/jphysiol.1975.sp010925 PMC1309459

[B128] Silinsky E. M. Redman R. S. (1996). Synchronous release of ATP and neurotransmitter within milliseconds of a motor nerve impulse in the frog. J. Physiol. 492, 815–822. 8734992 10.1113/jphysiol.1996.sp021348 PMC1158902

[B129] Siu P. M. Alway S. E. (2005). Mitochondria-associated apoptotic signalling in denervated rat skeletal muscle. J. Physiol. 565, 309–323. 10.1113/jphysiol.2004.081083 15774533 PMC1464486

[B130] Skaper S. D. (2012). The neurotrophin family of neurotrophic factors: an overview. Methods Mol. Biol. 846, 1–12. 10.1007/978-1-61779-536-7_1 22367796

[B173] Sköld C. Levi R. Seiger A. (1999). Spasticity after traumatic spinal cord injury: nature, severity and location. Arch. Phys. Med. Rehabil. 80, 1548–1557. 10.1016/s0003-9993(99)90329-5 10597805

[B169] Smith J. W. Thesleff S. (1976). Spontaneous activity in denervated mouse diaphragm muscle. J. Physiol. 257, 171–186. 948050 10.1113/jphysiol.1976.sp011362 PMC1309350

[B131] Song E. K. Rah S. Y. Lee Y. R. Yoo C. H. Kim Y. R. Yeom J. H.. (2011). Connexin-43 hemichannels mediate cyclic ADP-ribose generation and its Ca2+-mobilizing activity by NAD+/cyclic ADP-ribose transport. J. Biol. Chem. 286, 44480–44490. 10.1074/jbc.m111.307645 22033928 PMC3247979

[B132] Squecco R. Carraro U. Kern H. Pond A. Adami N. Biral D.. (2009). A subpopulation of rat muscle fibers maintains an assessable excitation-contraction coupling mechanism after long-standing denervation despite lost contractility. J. Neuropathol. Exp. Neurol. 68, 1256–1268. 10.1097/nen.0b013e3181c18416 19915489

[B133] Stadler H. Fenwick E. M. (1983). Cholinergic synaptic vesicles from Torpedo marmorata contain an atractyloside-binding protein related to the mitochondrial ADP/ATP carrier. Eur. J. Biochem. 136, 377–382. 10.1111/j.1432-1033.1983.tb07752.x 6313364

[B134] Stout C. E. Costantin J. L. Naus C. C. Charles A. C. (2002). Intercellular calcium signaling in astrocytes via ATP release through connexin hemichannels. J. Biol. Chem. 277, 10482–10488. 10.1074/jbc.m109902200 11790776

[B135] Tews D. S. (2002). Apoptosis and muscle fibre loss in neuromuscular disorders. Neuromuscul. Disord. 12, 613–622. 10.1016/s0960-8966(02)00030-5 12207928

[B162] Thesleff S. (1963). “Spontaneous electrical activity in denervated rat skeletal muscle,” in In The Effect of Use and Disse on Neuromuwcular Functions, eds Gutmann E. Hnik P. (Prague: Czechoslovak Academy of Sciences), 41–62.

[B137] Thomas S. A. Hume R. I. (1993). Single potassium channel currents activated by extracellular ATP in developing chick skeletal muscle: a role for second messengers. J. Neurophysiol. 69, 1556–1566. 8389829 10.1152/jn.1993.69.5.1556

[B136] Thomas C. K. Zaidner E. Y. Calancie B. Broton J. G. Bigland-Ritchie B. R. (1997). Muscle weakness, paralysis and atrophy after human cervical spinal cord injury. Exp. Neurol. 148, 414–423. 10.1006/exnr.1997.6690 9417821

[B182] Tisdale M. J. (2002). Cachexia in cancer patients. Nat. Rev. Cancer 2, 862–871. 10.1038/nrc927 12415256

[B138] Tomanek R. J. Lund D. D. (1973). Degeneration of different types of skeletal muscle fibres. I. Denervation. J. Anat. 116, 395–407. 4275502 PMC1271574

[B139] Tomanek R. J. Lund D. D. (1974). Degeneration of different types of skeletal muscle fibres. II. Immobilization. J. Anat. 118, 531–541. 4281422 PMC1231551

[B140] Tsuneki H. Salas R. Dani J. A. (2003). Mouse muscle denervation increases expression of an alpha7 nicotinic receptor with unusual pharmacology. J. Physiol. 547, 169–179. 10.1111/j..2002.00169.x 12562921 PMC2342616

[B141] Ung R. V. Rouleau P. Guertin P. A. (2010). Effects of co-administration of clenbuterol and testosterone propionate on skeletal muscle in paraplegic mice. J. Neurotrauma 27, 1129–1142. 10.1089/neu.2009.1211 20482256

[B142] Vizi E. S. Nitahara K. Sato K. Sperlágh B. (2000). Stimulation-dependent release, breakdown and action of endogenous ATP in mouse hemidiaphragm preparation: the possible role of ATP in neuromuscular transmission. J. Auton. Nerv. Syst. 81, 278–284. 10.1016/s0165-1838(00)00129-6 10869732

[B143] Volknandt W. Zimmermann H. (1986). Acetylcholine, ATP and proteoglycan are common to synaptic vesicles isolated from the electric organs of electric eel and electric catfish as well as from rat diaphragm. J. Neurochem. 47, 1449–1462. 10.1111/j.1471-4159.1986.tb00778.x 3760871

[B144] von Maltzahn J. Euwens C. Willecke K. Söhl G. (2004). The novel mouse connexin39 gene is expressed in developing striated muscle fibers. J. Cell Sci. 117, 5381–5392. 10.1242/jcs.01413 15466892

[B145] Voss A. A. (2009). Extracellular ATP inhibits chloride channels in mature mammalian skeletal muscle by activating P2Y1 receptors. J. Physiol. 587, 5739–5752. 10.1113/jphysiol.2009.179275 19805741 PMC2805382

[B146] Wallraff A. Köhling R. Heinemann U. Theis M. Willecke K. Steinhäuser C. (2006). The impact of astrocytic gap junctional coupling on potassium buffering in the hippocampus. J. Neurosci. 26, 5438–5447. 10.1523/jneurosci.0037-06.2006 16707796 PMC6675300

[B147] Ware F. Jr. Bennett A. L. Mc I. A. R. (1954). Membrane resting potential of denervated mammalian skeletal muscle measured in vivo. Am. J. Physiol. 177, 115–118. 13148353 10.1152/ajplegacy.1954.177.1.115

[B148] Weatherbee S. D. Anderson K. V. Niswander L. A. (2006). LDL-receptor-related protein 4 is crucial for formation of the neuromuscular junction. Development 133, 4993–5000. 10.1242/dev.02696 17119023

[B149] Williams A. H. Valdez G. Moresi V. Qi X. McAnally J. Elliott J. L.. (2009). MicroRNA-206 delays ALS progression and promotes regeneration of neuromuscular synapses in mice. Science 326, 1549–1554. 10.1126/science.1181046 20007902 PMC2796560

[B150] Wu X. Baer L. A. Wolf S. E. Wade C. E. Walters T. J. (2010). The impact of muscle disuse on muscle atrophy in severely burned rats. J. Surg. Res. 164, e243–e251. 10.1016/j.jss.2010.08.032 20888588 PMC2991603

[B151] Wu Y. Collier L. Qin W. Creasey G. Bauman W. A. Jarvis J.. (2013). Electrical stimulation modulates Wnt signaling and regulates genes for the motor endplate and calcium binding in muscle of rats with spinal cord transection. BMC Neurosci. 14:81. 10.1186/1471-2202-14-81 23914941 PMC3735397

[B152] Wu Y. Zhao J. Zhao W. Pan J. Bauman W. A. Cardozo C. P. (2012). Nandrolone normalizes determinants of muscle mass and fiber type after spinal cord injury. J. Neurotrauma 29, 1663–1675. 10.1089/neu.2011.2203 22208735 PMC5364642

[B154] Yang X. Arber S. William C. Li L. Tanabe Y. Jessell T. M.. (2001). Patterning of muscle acetylcholine receptor gene expression in the absence of motor innervation. Neuron 30, 399–410. 10.1016/s0896-6273(01)00287-2 11395002

[B153] Yang J. Dominguez B. de Winter F. Gould T. W. Eriksson J. E. Lee K. F. (2011). Nestin negatively regulates postsynaptic differentiation of the neuromuscular synapse. Nat. Neurosci. 14, 324–330. 10.1038/nn.2747 21278733 PMC3069133

[B155] Yang X. Li W. Prescott E. D. Burden S. J. Wang J. C. (2000). DNA topoisomerase IIbeta and neural development. Science 287, 131–134. 10.1126/science.287.5450.131 10615047

[B156] Ye Z. C. Wyeth M. S. Baltan-Tekkok S. Ransom B. R. (2003). Functional hemichannels in astrocytes: a novel mechanism of glutamate release. J. Neurosci. 23, 3588–3596. 12736329 10.1523/JNEUROSCI.23-09-03588.2003 PMC6742182

[B157] Zeman R. J. Zhao J. Zhang Y. Zhao W. Wen X. Wu Y. (2009). Differential skeletal muscle gene expression after upper or lower motor neuron transection. Pflugers Arch. 458, 525–535 10.1007/s00424-009-0643-5 19214561

[B158] Zhang W. Li H. Xing Z. Yuan H. Kindy M. S. Li Z. (2012). Expression of mRNAs for PPT, CGRP, NF-200 and MAP-2 in cocultures of dissociated DRG neurons and skeletal muscle cells in administration of NGF or NT-3. Folia Histochem. Cytobiol. 50, 312–318 10.5603/fhc.2012.0041 22763971 PMC3703251

[B176] Zhao C. Veltri K. Li S. Bain J. R. Fahnestock M. (2004). NGF, BDNF, NT-3 and GDNF mRNA expression in rat skeletal muscle following denervation and sensory protection. J. Neurotrauma 21, 1468–1478. 10.1089/neu.2004.21.1468 15672636

[B159] Zimmermann H. Denston C. R. (1976). Adenosine triphosphate in cholinergic vesicles isolated from the electric organ of Electrophorus electricus. Brain Res. 111, 365–376. 10.1016/0006-8993(76)90780-0 949609

